# Effect of Moisture Distribution Changes Induced by Different Cooking Temperature on Cooking Quality and Texture Properties of Noodles Made from Whole Tartary Buckwheat

**DOI:** 10.3390/foods10112543

**Published:** 2021-10-22

**Authors:** Shuping Zou, Lijuan Wang, Aili Wang, Qian Zhang, Zaigui Li, Ju Qiu

**Affiliations:** 1 College of Food Science and Nutritional Engineering, China Agricultural University, 17 Qinghua Dong Lu, Haidian District, Beijing 100083, China; 674111327@cau.edu.cn (S.Z.); lizg@cau.edu.cn (Z.L.); 2 Research Institute of Farm Products Storage and Processing, Xinjiang Academy of Agricultural Sciences, Urumqi 830091, China; xjnljzhq@xaas.ac.cn; 3 Department of Nutrition and Health, China Agricultural University, No.17 Qinghuadonglu, Haidian, Beijing 100083, China; wanglijuan@cau.edu.cn; 4 Key Laboratory of Coarse Cereal Processing, School of Food and Biological Engineering, Chengdu University, Chengdu 610106, China; wangaili@cdu.edu.cn

**Keywords:** cooking quality, cooking temperature, moisture distribution, whole Tartary buckwheat noodles

## Abstract

While precooking and processing have improved the quality of gluten-free noodles, the effects of different cooking temperatures on their quality—neither gluten-free noodles nor whole Tartary buckwheat noodles—have rarely been clarified. This study investigated the key role of moisture distribution induced by different cooking temperatures in improving the noodle quality of whole Tartary buckwheat. The results showed that cooking temperatures higher than 70 °C led to a sharp increase in cooking loss, flavonoid loss and the rate of broken noodles, as well as a sharp decrease in water absorption. Moreover, the noodles cooked at 70 °C showed the lowest rate of hardness and chewiness and the highest tensile strength of all cooking temperatures from 20 °C to 110 °C. The main positive attribute of noodles cooked at 70 °C might be their high uniform moisture distribution during cooking. Cooking at 70 °C for 12 min was determined as the best condition for the quality improvement of whole Tartary buckwheat noodles. This is the first study to illustrate the importance of cooking temperatures on the quality of Tartary buckwheat noodles. More consideration must also be given to the optimal cooking conditions for different gluten-free noodles made from minor coarse cereals.

## 1. Introduction

Noodles are popular worldwide as one of the major traditional staple foods, second only to bread consumption [1]. Approximately 40% of wheat in China is used for various types of noodle production [2]. Wheat noodles and rice noodles have been consumed for thousands of years because of their pleasant taste and flavor, but over-processing leads to a loss of nutrients, including dietary fiber, vitamins, minerals and phytochemicals, thus enabling the refined grains to become a possible cause of chronic disease [3,4]. Therefore, noodles made from whole grain or coarse cereals have become more popular within the last decade. Though not as commonly consumed around the world, they have significant health benefits induced by their enrichment in dietary fiber and other bioactive compounds [5]. However, it is not easy to make the noodle quality of coarse cereals comparable to that of wheat noodles because of the absence of gluten protein, which plays a key role in sensory quality and processing properties [6]. Thus, the processing optimization of gluten-free noodles has attracted growing attention from researchers and food manufacturers. 

Extrusion technology, which uses high temperatures, pressure and shear forces, is a feasible method for gluten-free noodle production. Extrusion processing is used widely to produce brown rice pasta, corn-broad beans pasta, and rice-yellow pea pasta [7,8,9]. Starch gelatinization is the main phenomenon exploited in the process of gluten-free noodle extrusion, and the different processing parameters (such as moisture content or temperature) affect the starch granule organization, which, in turn, affects the noodle quality [10]. However, few studies focus on the influence of cooking temperature on noodle quality after extrusion and just before eating, and there has been little proposed regarding the appropriate evaluation and improvement of cooking quality for gluten-free noodles made from whole coarse cereals. Taking into account the health benefits of Tartary buckwheat (*Fagopyrum tataricum*) rich in flavonoids and its past as a staple food in many Asian countries, such as Japan, China and Korea [7,11], it is necessary to explore effective ways to increase its dietary intake [12]. The extrude noodles made from whole Tartary buckwheat are an ideal way to go. Consequently, the investigation and application of different cooking conditions are critical in improving the cooking quality of noodles induced by gluten-free properties and special bioactive compounds to supply an effective method for food manufactures.

Given that the cooking quality of noodles made from Tartary buckwheat is distinct to wheat or rice noodles because of their differences in nutrient compositions and processing properties, clarification is needed regarding the effects of boiling prior to eating on the cooking quality of whole Tartary buckwheat noodles and their mechanism in water diffusion. This study aimed to: (1) investigate the optimum cooking temperature and time for an improved cooking quality and texture properties of extruded Tartary buckwheat noodles; (2) determine the changes in water distribution and migration during cooking to explain the mechanism; and (3) analyze the effects of the optimum cooking condition on the functional properties of Tartary buckwheat noodles, especially compared with the usual 100 °C boiling of other gluten-free noodles. 

## 2. Materials and Methods

### 2.1. Materials

The noodles made from 100% Tartary buckwheat were provided by Guiyang high-tech Yingna Technology Development Co., Ltd. (Guiyang, China). They were processed at 120 °C by a twin-screw extruder, according to previous methods [13]. The extrude noodles consisted of 81.43% starch, 8.0% protein, 1.40% dietary fiber and 8.89% moisture and were analyzed using standard procedures of the Association of Official Analytical Chemists. Rutin and gallic acid were purchased from Yuanye Biology Co., Ltd. (Shanghai, China). 

### 2.2. Determination of Optimum Cooking Time

Since the extruded noodles were ready to eat after soaking at room temperature, the temperature controller of the water bath was set up to be 20 °C, 30 °C, 40 °C, 50 °C, 60 °C, 70 °C, 80 °C, 90 °C, 100 °C and 110 °C, respectively. The Tartary buckwheat noodles were boiled in a thermostat water bath (noodles/water ratio was 1:20, *m*/*v*) with precise temperature controls ±2 °C (Heidolph company, Heidolph, Germany). The optimum cooking time of each temperature was determined by observing a brown core in the center of the noodles, which were taken out every 30 s during cooking, cut into 1 cm short strips and squeezed using a transparent glass plate. The time when the brown core disappeared was recorded as the optimum cooking time. 

### 2.3. Color of the Tartary Buckwheat Noodles

The cooked noodles were freeze-dried and milled through 60 mesh (HY-04A, Beijing Huanyatianyuan Instrument Co., Ltd., Beijing, China). The color of the cooked noodle powder was measured by a HunterLab colorimeter (DP-9000 D25A, Hunter Associates Laboratory, Reston, VA, USA) with a 40 mm aperture and a halogen tungsten lighting source lamp equipped with a C illuminant and 2° standard observer. The results were expressed as L* (lightness/darkness), a* (greenness/redness) and b* (blueness/yellowness) values. The instrument was calibrated with standard whiteboards and blackboards. Color value averages were calculated from triplicate measurements. The ΔE value representing the comprehensive value of the noodle was calculated as follows:

ΔE = [L*^2^ + a*^2^ + b*^2^]^1/2^

### 2.4. Cooking Qualities of the Tartary Buckwheat Noodles

The cooking qualities of Tartary buckwheat noodles, including water absorption, cooking loss and broken rate, were determined according to a reported method [14] with some modifications. Briefly, 25 g noodles were boiled in 500 mL water at different temperatures for optimum time. The cooking water was collected and filled up to 500 mL with distilled water. Cooking loss was measured by drying the cooking water at 105 °C for 12 h and expressed as the percentage of residues in the raw noodles. The water absorption was expressed as the mass ratio before and after cooking. The broken rate was the ratio of the number of broken noodles to that of dried noodles. All experiments were conducted in triplicate.

### 2.5. Texture Profiles of Tartary Buckwheat Noodles

Texture profile analysis (TPA) was conducted by a TA-XT2i Texture Analyzer with a cylindrical probe P/36 R (Stable Micro System Ltd., Surrey, UK), according to the reported method [15]. Twenty samples of Tartary buckwheat noodles were boiled at different temperatures with the optimum cooking time and cooled in cold water for 1 min, followed by the TPA test within 5 min. Cooked noodle strands (five strands per sample) measuring 10 cm were determined at the speed of 1 mm/s with a 50% compression ratio and a 5 g trigger force. The values of hardness, springiness and chewiness were recorded. The tensile test was conducted by the A/SPR probe at a test speed of 3 mm/s and a distance of 80 mm. The tensile force and tensile work were recorded. The shearing test of noodles was also conducted by the A/LKB-F probe with a diameter of 1 mm. The test speed was 0.17 mm/s, the return speed was 10 mm/s and the test distance was 5 mm. The maximum shear hardness was recorded as the hardness of the noodle. Each analysis was repeated six times. 

### 2.6. Total Flavonoid Content (TFC) and Total Phenolic Content (TPC)

The cooked noodle soup (0.5 mL) was mixed with 2 mL of distilled water and 0.15 mL of NaNO_2_ (5%) for 5 min, dissolved in 0.15 mL of AlCl_3_·6H_2_O (10%) for 5 min and then mixed with 1 mL of NaOH (1 mol/L) with the rest of 15 min. The absorbance of the mixture at 415 nm was measured by a spectrophotometer (T6, Beijing General Instrument Co., Ltd., Beijing, China) to determine TFC, which was calculated according to the standard rutin curve and expressed as a rutin equivalent (μg RE/g; dry weight). The concentration of rutin for the standard curve was 80, 160, 240, 320 and 400 μg/mL. The cooked noodle soup (0.5 mL) was mixed with 5 mL of Folin-Ciocalteu reagent (1 mol/L) and neutralized with 4 mL of saturated Na₂CO₃ (75 g/L) with a rest of 2 h. The absorbance at 765 nm was measured by spectrophotometer to determine TPC, which was expressed as a gallic acid equivalent (mg GAE/g; dry weight). The concentration of gallic acid for the standard curve was 10, 20, 30, 40, 50 and 60 μg/mL.

### 2.7. In Vitro Starch Digestibility

In vitro starch digestibility was measured according to the study of Goh et al. [16]. The cooked noodles (50 mg) homogenized in distilled water were mixed with 10 mL of HCl-KCl buffer (pH 1.5) and 0.2 mL of pepsin solution (0.1 g/mL) (P7000, from porcine gastric mucosa, 250 U mg^−1^, Sigma-Aldrich Chemical Co., St. Louis, MO, USA) in a shaking water bath at 40 °C for 1 h. Then, sodium acetate buffer (0.5 mol/L, pH 6.9) was added into the mixture and filled up to 25 mL, followed by the reaction with 5 mL α-amylase (2.6 IU) (10,080, from hog pancreas, 50 U mg^−1^, Sigma-Aldrich Chemical Co., St. Louis, MO, USA) in the water bath at 37 °C. The solution (1 mL) was taken out at 0, 15, 30, 45, 60, 90, 120 and 180 min, respectively, and the reaction was stopped in a 100 °C water bath for 5 min. The content of reducing sugar in the obtained solution was determined by DNS method, with glucose as the standard. The contents of rapidly digestible starch (RDS), slowly digestible starch (SDS) and resistant starch (RS) were defined as the fractions digested within 15 min, between 15 and 120 min and undigested after 120 min, respectively. The contents of RDS, SDS and RS were calculated according to reported equations [17].

### 2.8. Low-Field Nuclear Magnetic Resonance (LF-NMR) 

Moisture distribution in Tartary buckwheat noodles boiled at different temperatures was measured by LF-NMR (NMI20-030H-I, Niumag Analytical Instruments Co., Ltd., Suzhou, China), based on the published methods [18]. The transverse relaxations were measured using the Carr-Purcell-Meiboom-Gill (CPMG) sequence. CPMG decay curves were fitted to a multi-exponential function model using the MultiExpInvanalysis software (Suzhou Niumag Analytical Instrument Corporation, Suzhou, China). The parameters were as follows: echo time (TE) = 0.100 ms; the interval time of sampling (TW) = 1500 ms; scanning frequency (SF) = 22 MHz; and NECH = 1000. NMImaging_ver1.03 imaging software was used to determine the moisture distribution. The setting parameters were as follows: TE (ms) = 20.00; flip angle = 90°; refoc flip angle = 180°; averages = 2; read size = 256; RG (dB) = 20; TR (ms) = 500; TI-IR (ms) = 20; and phase size = 192. Time constants of the peak position were sequenced and recorded as T21 and T23, corresponding to bound water and free water, respectively. The amount of water with a different status could be calculated by the corresponding peak area. The percentage of the total area of each peak signified the relative content of the different status water marked as P21 and P23, respectively. 

### 2.9. Microscopic Morphology

The image of moisture distribution and migration in cooked noodles at different cooking temperatures was observed by a stereomicroscope (semidry-milled Z800, Nikon, Japan). The cooked noodles were cut into 1 cm length and observed directly. Before cooking, the noodles were also observed as controls to determine the brown core changes in their center. 

### 2.10. Statistical Analysis

The data were expressed as their mean ± standard deviations (SD) and analyzed by SPSS (Version 12.0 for Windows, SPSS Inc., Chicago, IL, USA). The significant differences were determined at *p* < 0.05 by one-way analysis of variance (ANOVA) and Tukey–Kramer’s multiple comparison post-hoc test. The correlations among the variables were determined by the two-tailed Pearson correlation analysis (*p* < 0.01). PCA was performed using Origin software (version 2019, Microcal Inc., Northampton, MA, USA). 

## 3. Results and Discussion 

### 3.1. Optimum Time at Different Cooking Temperatures

The cooking times of Tartary buckwheat noodles cooked at different temperatures were optimized (Table 1). A significant decrease in optimum cooking time was observed with an increase in cooking temperature. The whole Tartary buckwheat noodles were produced at 120 °C by a twin-screw extruder, enabling starch to be pregelatinized appropriately in the present study. This was similar to the previous report of extruded buckwheat noodles, in which the degree of gelatinization was around 70% [13]. The noodles could be cooked to eat in a water bath at temperatures lower than 100 °C, even at a room temperature of 20 °C. The immersion of extruded noodles in water was always the main cooking method for ready-to-eat gluten-free noodles, such as those made from rice, corn, potato and soy, because starch had been gelatinized during extrusion and these noodles were easy to break when boiling at 100 °C [10]. However, the immersion time of Tartary buckwheat noodles in water at 20 °C was 57 min, which was almost 9-fold longer than that of 100 °C. The optimum cooking time was longer than 20 min when the cooking temperature was lower than 50 °C, while the optimum cooking times at 60 °C or 70 °C were around 2-fold longer than that at 100 °C. This result indicated that the cooking time changed significantly with the cooking temperature for the full gelatinization of starch in extruded noodles, which further permanently affected noodle quality. For this reason, cooking temperatures lower than 100 °C with appropriate cooking times ought to be considered in subsequent studies. 

### 3.2. Effect of Cooking Temperature on Color and Cooking Qualities 

**Figure 1:**
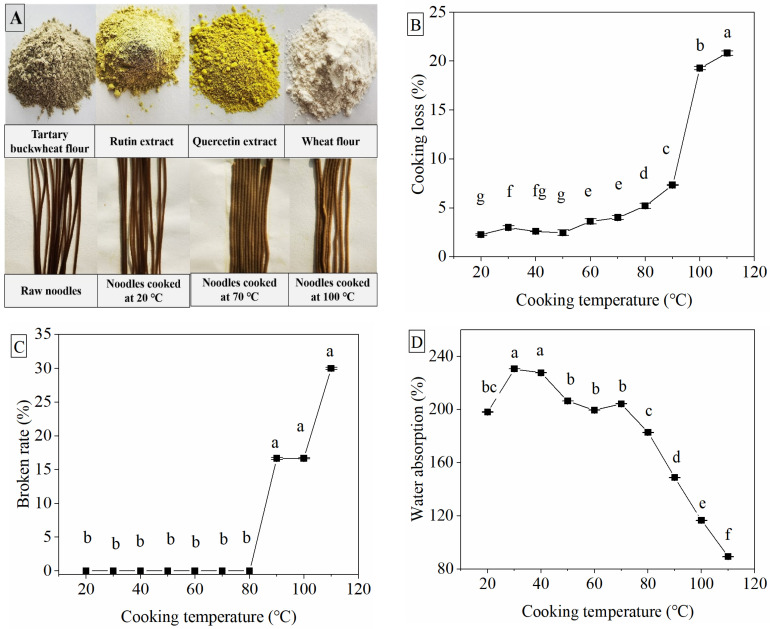
Cooking qualities of Tartary buckwheat noodles at different cooking temperatures. Typical images of noodle color and the color of rutin or quercetin extract (**A**), cooking loss (**B**), broken rate (**C**) and water absorption (**D**). Results are expressed by mean ± standard deviation (*n* = 3). Different letters indicate significant differences at *p* < 0.05.

Since the optimal color of Tartary buckwheat noodles was different to the white color of traditional wheat noodles, it was defined by the changes in values of L*, a* and b*. The color of the noodles was one of the most important quality parameters (Table 1, Figure 1A). A significant decrease in the L*, b* and ΔE values of noodles was observed until the cooking temperature was increased to more than 70 °C, and both L* and ΔE values were lowest at 100 °C and 110 °C out of all the cooking temperatures (*p* < 0.05) (Table 1). Meanwhile, an increase in a* value resulted from 70 °C cooking, and the highest a* value was also observed at 100 °C and 110 °C (*p* < 0.05). The L* value, representing brightness, indicated the color of cooked noodles and became brighter with the higher cooking temperatures (Figure 1A). The a* value, representing redness (from −green to +red), and b* value, representing yellowness (from −blue to +yellow), indicated the color of cooked noodles becoming less green and less yellow along with the increase in cooking temperature [19]. This might be attributed to the loss of nutrients such as flavonoids or phenolic acids—which is in line with other reports indicating that pigments such as quercetin and rutin in noodles or flour might leach out into the water during boiling, thus increasing redness and decreasing yellowness in boiling noodles [19,20]. The original color of Tartary buckwheat flour is green because of the richness in flavonoids mainly including rutin and quercetin (Figure 1A). The color of noodles becomes dark green because of the heat treatment with extrusion. Since the yellow and green colors represent the contents of the bioactive compounds of rutin and quercetin, the dark color should be representative of the good characteristics of whole Tartary buckwheat noodles, rather than the traditional color evaluation of wheat or rice noodles [21]. The corresponding loss of flavonoids or polyphenols was determined in later experiments.

There was an increase in the cooking loss of noodles at 60 °C and 70 °C, but a much sharper increase in cooking loss from 80 °C to 110 °C (Figure 1B). Little cooking loss represents a good cooking quality of noodles [22,23]. Cooking temperatures higher than 80 °C were not good for the structural maintenance of Tartary buckwheat noodles, because they might potentially have damaged the noodles during the cooking process. This finding was consistent with the previous study on pasta, which found that the cooking loss of non-gluten noodles was easy to increase because of the weakened structure network during the cooking process [24]. A significant increase in the rate of broken noodles was observed when the cooking temperature was higher than 80 °C (Figure 1C). The study also reported that the cooking loss and broken noodle rate during the cooking process are mainly due to the dissolution of loosely bound gelatinized starch on the surface of noodles, which mainly depends on the strength of the retrograded starch network around the gelatinized starch [25]. The water absorption of noodles cooked at 30 °C or 40 °C was higher than that at 20 °C, while that from 50 °C to 80 °C was the same as the levels at 20 °C, but sharply decreased at temperatures higher than 80 °C (Figure 1D) (*p* < 0.05). Water absorption is the ability of noodles to hold and retain water, which is primarily governed by starch, fiber, protein compositions and the strength of the protein or starch network [24]. Since the starch in extruded noodles had been generalized and was subjected to the secondary gelatinization during cooking when the temperature reached up to 60 °C, the significant difference in cooking loss and water absorption among each cooking temperature could be attributed to the different degree of starch gelatinization. As we know, the starch of Tartary buckwheat used to be gelatinized appropriately when the cooking temperature was 60–80 °C, and much higher temperatures would lead to excessive gelatinization, causing the disintegration or dissolution of the starch structure [25,26]. This might be the reason why cooking at temperatures higher than 80 °C induced a sharp increase in cooking loss and a decrease in water absorption. The present results showed that cooking temperatures ranging from 50 °C to 80 °C could maintain the same level of water absorption that noodles cooked at lower temperatures of 20 °C or below have, which might be appropriate for water distribution or the water-holding capacity of noodles during cooking. Based on these results, we determined that high levels of water absorption were undesirable for buckwheat noodles [26]. The sharp decrease in water absorption when the cooking temperature was higher than 80 °C may be attributed to the water volatilization at high temperatures and, thus, less water-holding capacity. Therefore, we detected the water distribution and migration of noodles next. 

### 3.3. Changes in Texture Profile Analysis of Noodles

Textural property is one of the major attributes influencing the overall quality of noodles. The TPA and tensile properties of noodles changed noticeably with the increase in cooking temperature (Table 2). The noodle showed the lowest rates of hardness and chewiness—but not springiness—when cooked at 70 °C. Hardness and chewiness are usually regarded as key indicators of the overall texture of noodles [13]. These parameters increased with the escalation in cooking temperature, from 20 °C to 70 °C, but decreased at 70 °C to 110 °C. The turning point at 70 °C indicated significant changes in the texture of the noodles. The texture properties of noodles are closely related to the compositions of starch, gluten and dietary fiber, which commonly determine the gel characteristics of noodles. As for wheat noodles, gluten contributes to the main gel characteristics, while for gluten-free buckwheat noodles, starch plays a key role in gel generation. A good noodle dependent on gluten gel usually appears firm, elastic, chewy and not overly sticky [1,17]. However, gluten-free noodles dependent on starch gel always result in the noodles being too hard to cook or eat, especially the extruded noodles made from whole grain without gluten, because of the starch gelatinization and aging in the process of extrusion. It has been reported that the hardness of gluten-free spaghetti or pasta is much higher than that of wheat noodles, and the lower levels of hardness and chewiness in gluten-free pasta may prevent structure disintegration with the lower cooking loss [27,28]. The present study also found this improvement in extruded noodles made from whole Tartary buckwheat by cooking at 70 °C, according to the lowest levels of hardness and chewiness together with a relative lower cooking loss. 

The tensile properties of noodles cooked at 70 °C showed higher values of tensile force and tensile work than those of noodles cooked higher than 90 °C or lower than 40 °C. This indicated that, compared with other cooking temperatures, the noodles cooked at 70 °C were not easily broken, even though their hardness and chewiness were at the lowest levels. The tensile force of noodles was reported to reflect elasticity and ductility [22], which supported the better elasticity and ductility of Tartary buckwheat noodles cooked specifically at 70 °C. The higher levels of tensile force and tensile work were responsible for the lower rate of broken noodles. The shear hardness of noodles increased with an increase in cooking temperature. These results suggest that the noodles cooked at 70 °C obtained the best cooking qualities, similar to the results of cooking qualities and color mentioned above. Moreover, these findings support that the better tensile properties and the relative lower hardness might be adapted to evaluate the cooking quality of whole Tartary buckwheat noodles. In addition, the texture properties were also relevant to the starch gelatinization or the water distribution during the secondary gelatinization, which is illustrated next.

### 3.4. Moisture Content and Water Distribution of Noodles during Cooking

**Figure 2:**
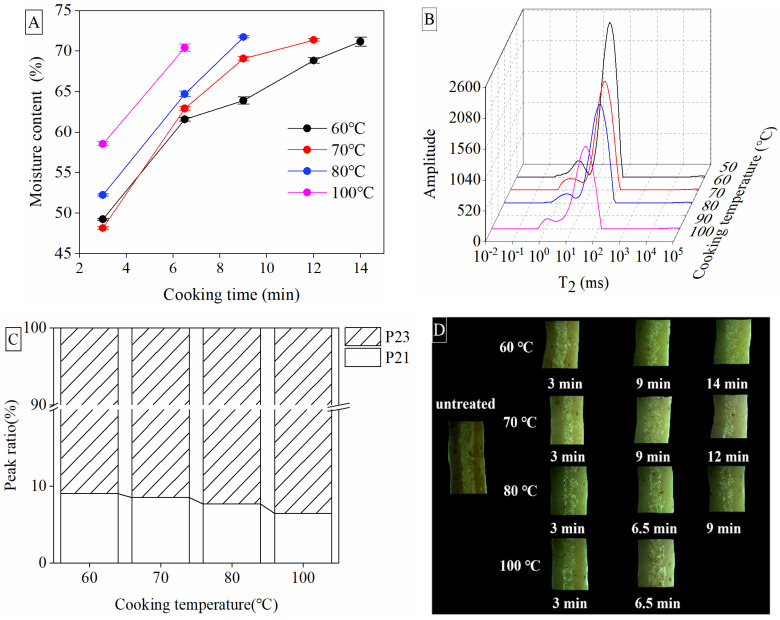
Water distribution and migration of Tartary buckwheat noodles. Moisture content (**A**); the comparison of T2 relaxation time at different cooking temperatures under the optimum cooking time (**B**); the comparison of T2 peak ratio (**C**); and microscopic images of noodles before and after cooking at different temperatures (**D**).

The differences in cooking quality and texture properties among different cooking temperatures were explained by the subsequent determination of water distribution and migration in noodles (Figure 2). Taking into account the above results of cooking qualities and TPA, together with the shorter cooking time, the optimum cooking temperature of 70 °C was compared with 60, 80 and 100 °C to illustrate the improvement in noodle quality. As shown in Figure 2A, the lower temperature and the longer cooking time were required to reach the same moisture content. The optimum cooking time of 60 °C, 70 °C, 80 °C and 100 °C was 14 min, 12 min, 9 min and 6.5 min, respectively, when the moisture content reached the same level around 70%. The transverse relaxation time (T2) of these different optimum cooking conditions was shown in Figure 2B. In general, short T2 indicates the close bond between the water and non-water components in noodles, while long T2 indicates more free water [29]. The present study showed two different water phases, including bound water expressed as T21 (0.1–10 ms) and free water as T23 (10–100 ms). The peak time of T21 of noodles cooked at high temperatures (80 °C or 100 °C, at 2.154 ms) was longer than that at low temperatures (60 °C or 70 °C, at 1.556 ms), which might indicate that low temperature leads to the more bound water with a slower speed of water migration. Since T21 data could reflect the mobility of water molecules in the matrix of swelling starches and the gluten network [30], cooking noodles at 60 °C or 70 °C probably better embedded the water molecules into the matrix of starches or the protein network than cooking at 80 °C or 100 °C. 

In addition, the peak amplitude of T21 and T23 changed significantly with the cooking temperature under each optimum cooking time (Figure 2B). Together with this, the P23 calculated by the integral area of T2 intervals escalated with the increase in temperature. There was more free water in noodles cooked at 100 °C (93.53%) than at 70 °C (91.50%), with less bound water (6.47% of 100 °C vs. 8.50% of 70 °C) (Figure 2C). This supported the theory that the low cooking temperature promoted more conversion from free water to bound water than the high cooking temperature when their moisture content was the same under each optimum cooking time. Namely, there was better water absorption of noodles cooked at 60 °C and 70 °C than at 80 °C and 100 °C, as mentioned above (Figure 1C). Bustos et al. [31] reported that the increase in water absorption with the hydration property of spaghetti was associated with starch swelling and gelatinization. Similarly to spaghetti, the starch in whole Tartary buckwheat noodles is already gelatinized, which means the noodles are edible even while soaked in water at room temperature as long as the water is fully absorbed by the noodles. 

The mobility and migration of water in noodles during different cooking processes were further confirmed by microscopic observation (Figure 2D). Before cooking, the starch structure of dry noodles was compact, showing full gelatinization of the peripheral starch, with partial gelatinization in the center and an apparent hard core. The water diffused gradually to the core of the noodles with the longer cooking time, and the hard core disappeared completely at the optimum cooking time of each temperature, compared with the uncooked noodles. The mobility speed increased causally with the increase in cooking temperature when cooking for 3 min. However, the water distribution was much more uniform in the noodles cooked at 60 °C for 14 min and 70 °C for 12 min, and the starch crystalline in the center was smaller than that of the noodles cooked at 100 °C for 6.5 min. The better water distribution of the noodles cooked at 70 °C explained why cooking at 70 °C showed the lowest rates of hardness, because the noodles softening during cooking would be accompanied by water migration from the surface to the core, along with the loss of firmness at the core. The same phenomenon was observed in a study on Japanese white salted noodles [30]. The study on the cooking quality of gluten-free noodles found that the speed of water migration to the center of the rice noodles was faster than that of spaghetti [32]. The present study also focused on the extruded noodles made from gluten-free ingredients of whole Tartary buckwheat noodles, which were similar to pasta. Their compact and dense structure resulted in a slower water diffusion than that of rice noodles during cooking, so that simply boiling water, which would result in an adequate taste in rice noodles, was not enough for Tartary buckwheat noodles to diffuse or distribute water uniformly. However, cooking at 70 °C with slow water diffusion was conducive to more uniform water distribution, fuller starch gelatinization and a better taste than the standard boiling at 100 °C. 

### 3.5. Effect of Cooking Temperature on Functional Properties

**Figure 3:**
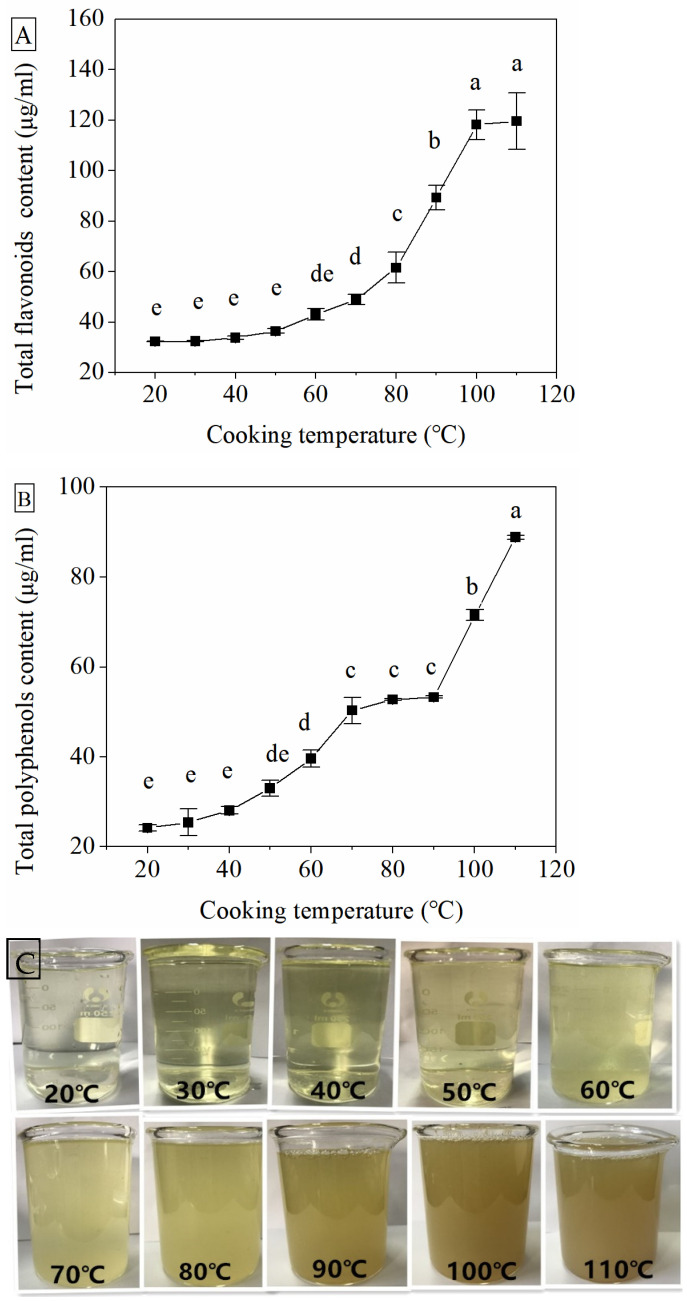
Total contents of flavonoids (**A**) and phenolics (**B**) in Tartary buckwheat noodle soup. Typical images showing color and turbidity of noodle soup after cooking (**C**). Results are expressed by mean ± standard deviation (*n* = 3). Different letters indicate significant differences at *p* < 0.05.

The nutrient content loss of flavonoids (TFC) and phenolics (TPC)—as the most famous bioactive compounds in Tartary buckwheat—was investigated (Figure 3). The significant increases in TFC and TPC in noodle soup were observed with the increased cooking temperature, especially the sharp increase resulting from cooking at temperatures higher than 80 °C (Figure 3A,B). The dark and turbid images of noodle soup were also clearly shown when cooking at temperatures higher than 80 °C (Figure 3C), which corresponded with the decrease in brightness and green and yellow colors. This indicated the nutrient loss of flavonoids and phenolics from noodles, which escalated with the increase in cooking temperature. The nutrient loss was also responsible for the more vibrant green and yellow colors based on the increase in b* and the decrease in a*, as well as increased brightness based on the increase in L* and ΔE values when the noodles were cooked at lower than 80 °C, as mentioned above. This finding was consistent with the previous studies on buckwheat pasta and rice-yellow pea pasta [9,33]. Since flavonoids and phenolics are reported with many bioactivities, such as antioxidative, anticarcinogenic, anti-inflammatory and prevention of various chronic diseases, their maintenance is necessary during cooking [34]. Therefore, cooking noodles at lower than 80 °C is an adequate way to protect the flavonoids and phenolics in Tartary buckwheat from loss.

**Figure 4:**
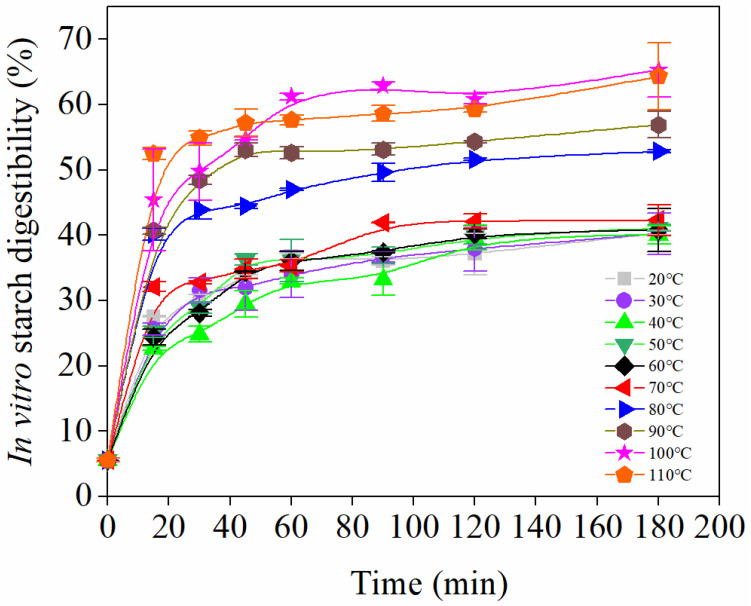
Hydrolysis kinetic curves of starch from Tartary buckwheat noodles cooked at different temperatures.

The cooking quality and water distribution affected the starch digestibility directly. It was reported that the faster the water diffusion, the greater and faster the gelatinization of starch, and thus the higher starch digestibility was obtained [35]. Similar to the findings in this study, the in vitro kinetics model of starch hydrolysis separated the starch digestibility of 20–70 °C from that of 80–110 °C clearly (Figure 4). There was no significant difference in starch digestibility among 20–70 °C (40.06–42.32%) at 180 min, but cooking at 100 °C (65.38%) and 110 °C (64.35%) showed the highest level of digestibility. This might be attributed to the fullest and fastest starch gelatinization of noodles cooked at 100 °C or 110 °C. It is worth noting that starch digestibility increased sharply within 45 min when cooking at temperatures higher than 70 °C, as well as increasing sharply within 60 min when cooking at lower than 70 °C, before changing gently afterwards. This turning point was 45 min into the study on rice-buckwheat noodles [22]. It indicated that higher cooking temperatures brought forward the time to reach the highest digestibility level. These findings highlighted the significantly lower starch digestibility of Tartary buckwheat noodles cooked at 70 °C compared to the ones cooked at higher than 80 °C. 

### 3.6. Association of Cooking Temperature with Noodle Qualities and Functional Properties

**Figure 5:**
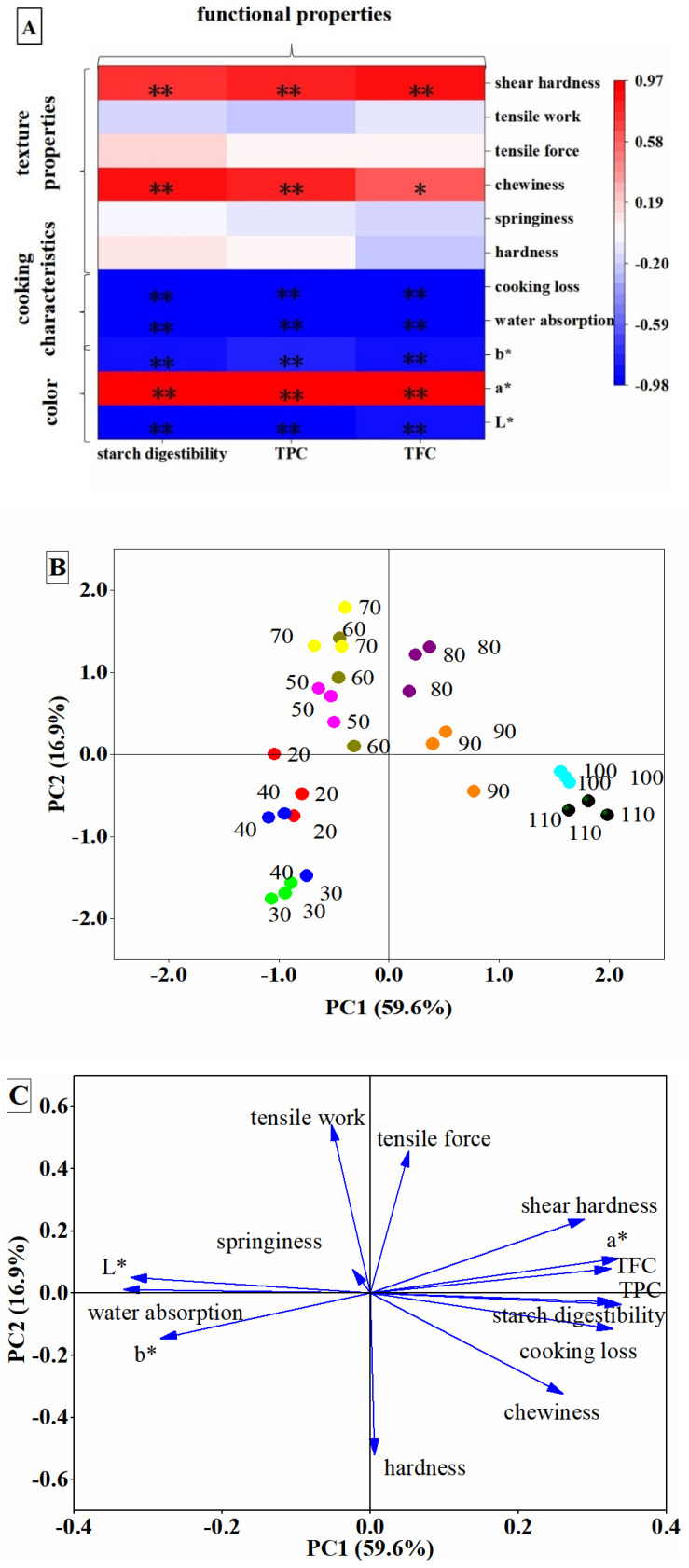
Association of cooking temperature with noodle quality. Pearson’s correlation of functional properties with cooking quality and texture profiles (**A**); a score plot of the noodles at different cooking temperatures (**B**); and a loading plot of the selected variables of noodles (**C**). Data with a single asterisk (*) and double asterisk (**) are statistically significant at *p* < 0.05 and *p* < 0.01, respectively.

The association of cooking temperature with noodle quality was analyzed (Figure 5A). The positive correlation of texture properties with functional properties indicated that the lowest level of chewiness, induced by cooking at 70 °C, corresponded with a lower amount of flavonoids and phenolics loss, as well as lower starch digestibility, while the highest shear hardness induced by cooking at 100 °C corresponded with more nutrient loss and higher starch digestibility. A correlation analysis between functional properties and color indicated that the darker the color of noodles, the lower the nutrient loss of flavonoids and phenolics was, as well as lower starch digestibility. The negative correlation of TFC, TPC or starch digestibility with cooking loss and water absorption indicated that nutrients were lost and starch became digestible during cooking. 

A PCA analysis explored and visualized the effect of cooking temperature on the quality and texture properties of noodles. The cumulative variance contribution rate of PC1 (59.6%) and PC2 (16.9%) was 76.5% (Figure 5B). Four clusters of cooking quality were separated clearly, including 20–40 °C, 50–70 °C, 80–90 °C and 100–110 °C. The loading plot supported the above results of noodle qualities that cooking at higher than 80 °C led to, including higher rates of starch digestibility, shear hardness, tensile force and TFC loss according to the positive sides of PC1 and PC2 (Figure 5C). Cooking at higher than 90 °C resulted in more cooking loss, chewiness, hardness and TPC loss according to the positive side of PC1 and the negative side of PC2. In terms of negative PC1 and positive PC2, 50–70 °C were predominantly affected by water absorption, tensile work and color value L. These results indicated that the change in the quality of noodles induced by cooking temperature was of great importance to functional properties, especially the difference in water distribution and migration. 

## 4. Conclusions

The present study illustrated that cooking at 70 °C for 12 min was the best condition to obtain a lower cooking loss, broken rate and higher water absorption of the noodles made from whole Tartary buckwheat, as well as lower hardness with a higher tensile force. The more improved quality of noodles cooked at 70 °C, rather than at the usual 100 °C, could be attributed to both the appropriate starch gelatinization and the relative slower water diffusion together with more uniform distribution, corresponding with less nutrient loss and lower starch digestibility. The present finding proposes the novel evaluation standards of TPA for the extruded noodles made from whole Tartary buckwheat. The novel way to control the cooking temperature of the whole coarse cereals’ noodles will be discussed in future studies.

## Figures and Tables

**Table 1:** Effect of temperature on optimal cooking times and the color of Tartary buckwheat noodles.

Cooking Temperature/°C	Optimal Cooking Time/min	Color			
		L*	a*	b*	ΔE
20	57.00	62.37 ± 0.61 ^a^	0.16 ± 0.06 ^f^	14.49 ± 0.58 ^a^	64.03 ± 0.85 ^a^
30	41.00	60.95 ± 0.28 ^a,b^	−0.14 ± 0.05 ^g^	13.94 ± 1.05 ^a,b^	62.52 ± 1.09 ^a,b,c^
40	27.50	61.46 ± 0.91 ^a^	0.10 ± 0.05 ^f^	14.32 ± 1.18 ^a,b^	63.11 ± 1.49 ^a,b^
50	18.00	59.70 ± 0.18 ^b,c^	0.37 ± 0.08 ^e^	12.38 ± 1.02 ^b^	60.97 ± 1.08 ^c,d^
60	14.00	61.04 ± 1.00 ^a,b^	0.36 ± 0.06 ^e^	12.36 ± 1.43 ^b^	62.28 ± 1.75 ^b,c^
70	12.00	61.34 ± 0.75 ^a^	0.53 ± 0.19 ^d^	12.65 ± 1.27 ^a,b^	62.63 ± 1.48 ^a,b^
80	9.00	58.94 ± 1.12 ^c^	0.74 ± 0.03 ^c^	9.99 ± 0.85 ^c^	59.79 ± 1.41 ^d,e^
90	7.50	58.35 ± 0.36 ^c^	0.91 ± 0.06 ^b^	10.37 ± 1.39 ^c^	59.27 ± 1.44 ^e^
100	6.50	55.04 ± 0.59 ^d^	1.39 ± 0.10 ^a^	10.31 ± 1.07 ^c^	56.01 ± 1.23 ^f^
110	5.50	55.75 ± 1.08 ^d^	1.40 ± 0.05 ^a^	9.71 ± 0.22 ^c^	56.60 ± 1.09 ^f^

Data are expressed by mean ± standard deviation and the different letters in the same column indicate significant differences (*p* < 0.05).

**Table 2:** Effect of cooking temperature on texture properties of noodles.

Cooking Temperature/°C	TPA			Tensile Properties		Shear Characteristics
	Hardness /N	Springiness /%	Chewiness /N	Tensile Force/N	Tensile Work/N*s	Shear Hardness/N
20	65.45 ± 4.19 ^a,b^	89.55 ± 2.09 ^a^	29.12 ± 2.27 ^c,d^	0.10 ± 0.00 ^b,c^	4.53 ± 0.72 ^d^	2.55± 0.17 ^f^
30	66.95 ± 1.42 ^a^	88.24 ± 0.58 ^a^	28.93 ± 0.44 ^c,d^	0.07 ± 0.01 ^f^	1.96 ± 0.29 ^f^	2.32 ± 0.28 ^f^
40	60.55 ± 7.32 ^a,b,c^	86.44 ± 5.36 ^a^	27.94 ± 4.25 ^c,d^	0.07 ± 0.00 ^e,f^	2.81 ±0.21 ^e,f^	2.64 ± 0.05 ^f^
50	60.41 ± 0.91 ^a,b,c^	84.41 ± 2.34 ^a^	26.49 ± 1.41 ^d,e^	0.09 ± 0.01 ^b,c^	7.30 ± 0.47 ^a^	3.63 ± 0.15 ^d,e^
60	46.07 ± 5.33 ^d^	89.83 ± 0.78 ^a^	26.64 ± 2.00 ^d,e^	0.08 ± 0.00 ^b,c,d^	4.45 ±1.18 ^d^	4.61 ± 0.24 ^b^
70	43.58 ± 1.83 ^d^	88.16 ± 0.72 ^a^	23.51 ± 0.51 ^e^	0.09 ± 0.01 ^b,c^	6.19 ± 0.44 ^b^	3.51 ± 0.03 ^e^
80	55.16 ± 2.63 ^c^	91.46 ± 1.45 ^a^	29.38 ± 0.90 ^c,d^	0.11 ± 0.01 ^a^	5.54 ±0.06 ^b,c^	4.22 ± 0.20 ^c^
90	58.67 ± 5.85 ^b,c^	90.90 ± 1.94 ^a^	31.18 ± 2.18 ^b,c^	0.08 ± 0.01 ^c,d,e^	4.95 ± 0.35 ^c,d^	3.94 ± 0.04 ^c,d^
100	59.50 ± 0.78 ^b,c^	87.44 ± 0.32 ^a^	35.30 ± 1.24 ^a^	0.10 ± 0.01 ^b^	3.50 ±0.48 ^e^	4.64 ± 0.22 ^b^
110	60.14 ± 0.64 ^a,b,c^	84.99 ± 1.84 ^a^	33.81 ± 1.97 ^a,b^	0.07 ± 0.00 ^d,e,f^	2.90 ± 0.05 ^e,f^	5.38 ± 0.26 ^a^

Values are expressed by mean ± standard deviation and the different letters in the same column indicate significant differences (*p* < 0.05).
